# Materia Medica and Therapeutics

**Published:** 1858-01

**Authors:** 


### MATERIA MEDICA AND THERAPEUTICS.

1. ON THE ACTION OF COD-LIVER OIL IN CHEST DISEASES.—[At a recent meeting of the Medical Society of London, *Dr. E. Smith* read a paper on the action of cod-liver oil, an abstract of which communication, together with the discussion that followed, we take from the *Lancet* of Nov. 7.]

The object of the paper was to show, that while cod-liver oil is a valuable remedy, it acts simply as a fat with certain physical qualities, and may be, and ought to be, supplanted by the ordinary fats used in food. The author pointed out the numerous and serious evils which result from public patronage of this remedy by the profession as regards the relation which exists between the public and the profession, and the great expense imposed upon our charities and poor-law surgeons. He believed that it has tended to lessen the gains of the profession by the indiscriminate employment of it by the public without professional advice, and has tended greatly to lessen the respect for the knowledge of the profession which it is the interest of both the profession and the public to maintain, and, in fact, that it has become, under the especial patronage of medical men, the great quack medicine of the day.

He first considered the chemical constitution of the oil, and then the state of system existing in phthisis, and particularly entered into an inquiry which he had instituted upon five hundred cases as to the appetite for the various fats in food, and the appetite for and effects of the oil. He found that there was a diminished appetite for fat in phthisis, but that in no case was the dislike absolute; and in all some kinds of fat could be taken when others were rejected. Thus, fat meat was taken by more than half of the patients, and bacon fat by two-thirds; while suet in puddings, butter, and milk, was taken by more than nine-tenths of the whole. Afterward he stated the result of the effect of cod-liver oil upon one hundred and fifty in-patients, and proved that in nearly half of them the oil was rejected or of no real service. Of eighty-three who reported themselves benefited, by far the larger proportion referred the good effect to improved assimilation; while of sixty-seven who were unable to continue its use, or who were not benefited, the ill effect was referred to sickness and other circumstances connected with the first acts of digestion. After thus showing the degree of good and harm resulting from the oil, and the degree of appetite for and dislike of fat of various kinds in food, he proved that in the large majority of cases there was an appetite for fats when oil did good, and the contrary when it did harm, but this was not universal; and he also proved that in the very cases in which the oil was rejected there was always appetite for some kind of fat food, and in some cases even for the fat of meat. Hence he proved that there was no ground for the assertion that the oil was more easily digested and assimilated than some of the natural fats in food. The inquiry and medical testimony now prove that cod-liver oil acts beneficially as a nutrient; and he affirmed, from special experience, that the oil has no advantage over the fats in food, provided an increased quantity be taken of such of the nutrient fats as the patient can eat. Hence he urged the profession to distinguish properly between food and physic; and, if the oil is administered as a nutrient, to inform the patient that it is given as food, or, as he preferred, to direct them to increase the nutrient fats in proper quantities and of a suitable kind, and to administer other articles as medicine.

The following were the author's conclusions:—

That neither cod-liver oil nor fat of any kind removes phthisis.

That the cases of arrest of the disease are very few.

That commonly the disease progresses, and is as fatal now as it was before the oil came into general use.

That in about half of the cases the rate of progress is retarded.

That the patient may be both stronger and stouter, and yet the disease quietly progress.

That when the oil disagrees, it is chiefly from its influence upon the digestive organs.

That when it agrees, it is chiefly by improving nutrition; but that in many cases it is believed by the patient to have a local influence.

That this local influence is most important in the pharynx and other parts of the mucous tract.

That it acts almost entirely as a fat, and supplies a defect in the system.

That it has no advantage over fats used in food, and may, like some of them, be taken and rejected.

That there is a large class in whom it is not beneficial, and it should be used with discrimination.

That it is our duty and our interest to dissociate food and physic.

Lastly, that it leaves the essence of the disease untouched; but that the great good which it often does temporarily, proves the importance of fat in the animal economy.

He showed how much more frequently there is appetite for fat in bronchitis than in phthisis and mere debility, and particularly in liver diseases; but he deferred the discussion of the question, and of the use of fats in general, to another occasion. He urged upon the profession the impropriety of poor-law surgeons being compelled to supply the oil, when the guardians ought to give fat in food, and of the medical officers of hospitals and dispensaries taxing the overburdened funds to supply so expensive a food.

Dr. Payne Cotton remarked that the opinions of the author respecting the virtues of cod-liver oil were not those entertained by the other physicians of the hospital for consumption. He himself was an advocate for its employment in all cases. He did not pretend to explain why it did good, but that it was of benefit was sufficiently evident by its effects. Under its use a great number of patients improved, and though it failed in some instances, it did not do so so frequently as Dr. Smith stated. He could only say he had undiminished confidence in its effects. He had tried other oils with a view to determine whether they could be substituted for it; but they were all inferior to it. Among those he had thus tested were train oil, neat's-foot, almond, olive, and cocoanut oil. That which most nearly resembled the cod-liver oil was the neat's-foot. When cod-liver oil was given in the earlier stages of phthisis, in many cases it arrested and retarded the disease, and prolonged the life of the patient.

Dr. Parrott inquired if the cod-liver oil had been used endermically? and whether glycerine had been tried as a substitute for it?

Dr. Cotton had employed glycerine, but it did not act beneficially.

Dr. Semple considered, from the experience he had had of the use of cod-liver oil, that it was of signal benefit in cases of phthisis. In many cases it prolonged life, and added to the comfort of the patient: in some instances, when given in the early stages of the disease, it arrested the complaint and prolonged life to its average duration. He had witnessed remarkable good effects from its administration to children affected with various strumous diseases.

Dr. Webster regarded cod-liver oil as beneficial as an article of food, and that it only did good in phthisis by its nutritive qualities. Phthisis was as fatal now as formerly; and this seemed to show that cod-liver oil had no effect in curing the disease.

Mr. Streeter thought cod-liver oil acted as did other fatty food, except that it contained iodine, which gave it a peculiar property. He thought the profession had been led astray with respect to this medicine, and had trusted too much to it of late in cases of phthisis. He eulogized the use of the old spermaceti mixture, and the preparation of iron and zinc, in phthisis.

Dr. Hughes Willshire said that his experience was opposed to that of Dr. Smith respecting the virtues of cod-liver oil. Whether it acted as a simple nutrient or as a medicine, would depend on the school of pathology to which the person who gives his opinion belongs. In his opinion the oil was of great value in various diseases of children, and in phthisis.

Dr. Camps regarded cod-liver oil as a valuable medicine, and not simply as an article of diet.

2. OIL OF TANSEY.—*Dr. Chapin*, of Winchester, [at a recent meeting of the Middlesex East (Mass.) District Medical Society,] introduced the subject of oil of tansey in its ecbolic and toxicological relations, and related a case in which he was summoned at midnight to visit a married female "in a fit." The patient was found in bed, partly conscious, and in paroxysms. A distinct smell of the oil pervaded the apartment. Vomiting had occurred. He immediately exhibited ipecacuanha and sulphate of zinc, which was followed by free emesis. In an hour the mind became clear, and she got along very well. The woman was four months advanced in pregnancy, and took the oil for abortion. The quantity taken was half a fluidounce. Dr. Chapin stated that some cases have been fatal.

Drs. B. Cutter and Drew, of Woburn, adduced similar cases, and Dr. Underwood, of West Cambridge, spoke of a young woman in a hotel who took the oil to procure abortion. The immediate effect was violent convulsions. At full term a child was born, *not larger than a rat.* The child lived three weeks. This case was mentioned to show that the oil sometimes arrests growth. Dr. Toothaker, of Wilmington, spoke of a middle-aged married woman who took two fluidounces of oil of tansey in divided doses, without effect. She then resorted to the woods, although it was midwinter and the snow knee-deep, and gathered a quantity of savin leaves, an infusion of which was freely taken, without success. At term, she bore a medium-sized child, which for some time was esteemed *non compos.* Now, however, at the age of ten, the child is a bright boy.—*Boston Medical and Surgical Journal*, Dec. 10, 1857.

3. ARSENIC IN CHOLERA.—[As the present progress of cholera from the eastern toward the western parts of the European continent, and its more recent outbreak in the suburbs of London, render it not at all improbable that ere long we may again suffer from this terrible scourge, any new facts in regard to its treatment will prove valuable to our readers. The relief given to a number of cases occurring in London by the use of the arsenical solution in the hands of various practitioners has excited a great deal of interest, and the question in regard to the antidotal powers of the remedy is being much discussed in the British medical journals. As illustrative of the successful results obtained, we quote the three following cases, reported in the *Lancet* of Nov. 28, by C. BLACK, M.D., F.R.C.S., etc.]

The importance of arsenic in the treatment of cholera is a matter of such vital consequence to public health, and of such interest to the physician, in a therapeutical point of view, that it is my intention to place before the profession, from time to time, the results of my experience of this remedy in choleraic disease.

By the production, in this way, of a mass of incontrovertible data of its efficiency in cholera, it will, I trust, force itself upon the attention of medical men; and, as certainly as it shall do this, it will prove to them its specific power over the disease which has hitherto yielded such scanty results to the best-directed efforts of medical art. In placing this high value on the anti-choleraic virtue of arsenic, I yield, not to enthusiasm, but to experience—an experience which, at the present moment, embraces the treatment by this remedy of nearly two hundred cases, in none of which has the arsenic ever failed to produce a speedy and permanent cure. What other remedy, empirical or rational, has produced such results? It is certain that the records of medicine, so far as I know, do not furnish a parallel. If, indeed, the want of experience had lacked the justification of its adoption, the pathological views which are entertained by the majority, of the proximate cause of cholera, and the knowledge which physiological science has educed of the behavior of one poison in the presence of another within the human body, would have placed it in the category of rational, and not of empirical remedies. But experience has justified what theory sanctioned. Not only has it done this, but it will further show, in the details of the following cases, that the remedy in question is applicable to every phasis of choleraic disease, from that which threatens with death within the lapse of a few hours, to that which, by a slower process of passive drain from the bowels, consumes days in bringing its victim to a premature grave.

CASE I.—L. G——, aged forty-eight years, married, by trade a basket-maker, at six P.M. on September 22d, 1857, began to suffer from diarrhœa. From this hour until ten P.M. the bowels were moved four times, the evacuations being thin, watery, and offensive in odor. At ten the purging became much more frequent and severe, and was accompanied by almost incessant vomiting and cramps of the abdomen, legs, and even the muscles of the back of the neck. From the time above mentioned until five P.M. of the 23d, the patient is reported to have vomited and purged at least forty times. Shortly after this hour I saw him. He was writhing to and fro upon a bed, which was placed opposite his house-door, in a room ten feet by twelve, with a ceiling not more than six feet high. The apartment was lighted by a small window, which, together with another window of a foot square, and the door in question, afforded the only means of ventilating the whole house. His countenance was haggard and shrunken in the extreme; eyes hollow; nose and the parts around the mouth of a deep leaden hue; skin very cold; breath cold; voice hollow, squeaking and tubular; frequent thirst; pulse scarcely perceptible; breathing hurried and labored. He had not passed urine since four the night before. A constant moaning was heard, except on the return of cramps, which occurred every three or four minutes, when his sufferings excited loud cries of pain. The purging and vomiting were all but incessant, the vomits and dejections being copious, thin, and evidently serous in their character. He was ordered five drops of liquor arsenicalis every fifteen minutes until the symptoms became less urgent, and then to have the same dose every hour until my next visit.—Twelve A.M.: After the first dose of arsenic there was no return of cramps; the third dose was followed by a complete arrest of purging, while vomiting had occurred but three times during the interval of my first and second visits. The countenance still maintained a haggard expression; the nose in part its leaden hue; a slight degree of warmth was beginning to creep over the skin, and the pulse was now distinct at 120 per minute. Thirst was much less frequent; the breathing less hurried and labored; but neither had the voice lost its peculiar tone, nor had the secretion of the kidneys as yet been restored. He was ordered to take the usual dose of arsenic every hour.—Nine P.M.: The bowels were moved once at three P.M., the evacuations being scanty, and of increased consistence. Has vomited twice, but has experienced no return whatever of cramps. Skin warm; no secretion of urine; pulse 100, larger. To take three drops of the arsenical solution every third hour.

Sept. 24th.—Nine A.M.: Neither purging, vomiting, nor cramps since last visit. Skin hot; face flushed; thirst; pulse 96 per minute, full and strong; passed half an ounce of urine at six this morning for the first time since the commencement of the attack. To discontinue the further use of arsenic, and to take a mixture consisting of the acetate of ammonia, potassio-tartrate of antimony, spirit of nitrous ether, and water.

25th.—Quite well.

*Remarks.*—This case presented, in the time of its occurrence and the manifestation of its symptoms, all the characteristics of a malignant attack; yet five drops of the arsenical solution completely allayed the cramps, and fifteen the purging; while a few more drops placed the patient in perfect safety. Confident of the power of my remedy to control the disease, notwithstanding the extreme degree in which the case was when I first saw it, I neither ordered friction to be observed, nor the application of external heat to the body; nor did I administer or cause to be administered a single drop of any stimulant whatever. On the contrary, the door of the house was allowed to remain open, and a cold draught of air to play upon my patient, whose only drink consisted of a moderate supply of cold water. These apparent disadvantages I purposely incurred in order to test, as far as possible, the curative power of the arsenic. How well it answered my expectations, the easy, rapid, and complete recovery of the case amply testifies.

CASE II.—H. S——, aged forty-four, married, was seized with sickness and purging at noon, Sept. 24th, 1857. These symptoms did not become urgent until six o'clock in the evening, at which time they became more frequent, and were accompanied by occasional cramps of the abdomen. There were now, in addition to the above symptoms, coldness of the general surface, shivering, collapsed features, and thirst, but no cramps of the extremities, alteration of voice, or any leaden hue of the nose and lips. The pulse was 110 per minute, small, feeble, and stopped by the least pressure. She had not voided urine since twelve A.M. She was ordered to take five drops of the arsenical solution every half hour.

Ten P.M.—Has neither vomited nor purged since taking the first dose of arsenic. Says that she felt the remedy at once affect her for the better. Has now and then slight twinging pains in the muscles of the abdomen; feels comfortable; skin becoming warm; pulse 96, larger. To take three drops of the arsenical solution every second hour.

Sept. 25th.—Nine A.M.: Has passed a comfortable night; bowels moved once; neither nausea nor sickness; skin hot; tongue moist, but coated with a white fur; thirst; passed two ounces of urine early in the morning; pulse natural in frequency, but somewhat fuller and stronger. To discontinue the arsenic, and to take a simple diaphoretic mixture.

26th.—Nine A.M.: Neither purging nor vomiting since last visit; feels quite well.

CASE III.—H. R——, aged twenty-seven, son of the above, married, was suddenly seized. at nine P.M., Sept. 24th, 1857, with violent shivering, sensation of impending death, and complete inability to stand or walk. This occurring at a friend's house, he was carried home and immediately put to bed. In a few minutes afterward I saw him. He was then shivering violently beneath a load of blankets, was surrounded by bottles of hot water, and complained of an intolerable weight at the præcordia. The symptoms of collapse were well marked, and were becoming more and more so every minute. He was ordered to take six drops of the arsenical solution every twenty minutes or half-hour.—Ten P.M.: Immediately before taking the first dose, violent cramps of the abdomen had set in, and directly after the exhibition of that dose, a severe paroxysm of vomiting and purging occurred. The vomit consisted of a colorless, glairy, sero-mucoid fluid, mingled with small quantities of food. The dejection was thin, watery, of a faintly bilious tinge, and of a sickly odor. A second dose of arsenic, administered immediately after the rejection of the first, arrested both the vomiting and purging. Has now occasional twinging pains in the muscles of the abdomen and calves, but no decided cramps. The shivering and extreme coldness of skin have given place to a slight degree of warmth. Expresses himself as feeling much better; slight thirst; pulse 100, soft and regular. To continue the arsenic every second hour.

Sept. 25th.—Nine A.M.: Found him sitting up, and down stairs. Took the arsenic until the middle of last night, and then discontinued it, as he felt well. —Nine P.M.: At six this evening the sickness, purging, and cramps returned. He resumed the arsenic, and was relieved by the first dose. To continue it regularly every third hour during the night.

26th.—Nine A.M.: Has passed a comfortable night; neither purging, vomiting, nor cramps since last visit; no thirst; appetite improving; skin warm; pulse natural. To take two drops of the arsenical solution every fourth hour.

27th.—Discontinued the arsenic last evening. Quite well.

*Remarks.*—The above two cases occurred in the same house and on the same day. The former was one of cholera of ordinary occurrence. The latter, in its very outset, threatened to be unusually severe. In the former, the first dose of arsenic permanently arrested both vomiting and purging; in the latter, a similar result was obtained by a second dose. The latter case exemplifies, in a remarkable manner, the specific value of arsenic—firstly, by a recurrence of the choleraic symptoms on too early a discontinuance of the remedy; and, secondly, by the *immediate* control of those symptoms on the resumption of the arsenic.

